# 972. Imipenem-Cilastatin-Relebactam (I/R) Pharmacokinetics (PK) in Critically Ill Patients Supported on Extracorporeal Membrane Oxygenation (ECMO)

**DOI:** 10.1093/ofid/ofad500.027

**Published:** 2023-11-27

**Authors:** Andrew J Fratoni, Abigail Kois, Jason A Gluck, David P Nicolau, Joseph L Kuti

**Affiliations:** Hartford Hospital, Hartford, CT; Hartford Hospital, Hartford, CT; Hartford Hospital, Hartford, CT; Hartford Hospital, Hartford, CT; Hartford Hospital, Hartford, CT

## Abstract

**Background:**

ECMO provides life-saving cardiac and/or pulmonary support to critically ill patients, but its effects on the PK of many antimicrobials remain unknown. Herein, we describe the PK of imipenem (IMI) and relebactam (REL) in critically ill patients supported on ECMO.

**Methods:**

Patients with confirmed or suspected infections supported on veno-venous or veno-arterial ECMO received 4 doses of I/R based on estimated creatinine clearance (CrCL) in accordance with current prescribing recommendations. Blood samples were collected over 6 h after last dose. IMI and REL concentrations were determined by a validated LC/MS/MS assay. Protein binding was assessed by ultrafiltration. Population PK models with and without covariates were fit using the non-parametric adaptive grid algorithm in Pmetrics for R. A joint probability of target attainment (PTA) at steady state using REL *f*AUC/MIC ≥ 8 and IMI ≥ 40% *f*T >MIC was assessed using 1000 patient Monte Carlo simulations for each labeled dosing regimen across CrCL ranges of 15-29, 30-59, 60-89, and 90-130 mL/min.

**Results:**

Seven patients (5 males) on ECMO received I/R; 1 patient received a single dose of I/R. Mean ± SD age, weight, and ECMO flow rate were 50 ± 16 years, 119 ± 12 kg, and 4.2 ± 0.5 L/min, respectively. Median (range) CrCL were 113 (32-317) mL/min. A two-compartment population PK model using CrCL as a covariate on both IMI and REL CL [CL=CL_0_*(CrCL/113)] fitted the data best. ECMO flow rate did not influence IMI or REL CL. The mean ± SD parameters for IMI were: CL_0_, 15.21 ± 6.52 L/h; V_c_, 10.13 ± 2.26 L; K_12_, 2.45 ± 1.16 h^-1^; and K_21_, 1.76 ± 0.49 h^-1^. REL PK parameters were 6.95 ± 1.34 L/h, 9.81 ± 2.69 L, 2.43 ± 1.13 h^-1^, and 1.52 ± 0.67 h^-1^, respectively. Protein binding for both drugs was 0%. I/R at currently labeled dosing regimens achieved > 90% PTA at the MIC of 2/4 mg/L for CrCL between 15-130 mL/min, supporting efficacy against both Enterobacterales and *Pseudomonas aeruginosa*.

**Conclusion:**

These are the first data to describe I/R PK in critically ill patients supported by ECMO. For patients treated with ECMO, currently labeled dosing regimens of I/R guided by estimated CrCL are predicted to achieve optimal exposures necessary to treat susceptible Enterobacterales and *P. aeruginosa* isolates.

**Disclosures:**

**Abigail Kois, PharmD**, AstraZeneca: Sr scientist, clinical pharmacology R&I|AstraZeneca: Stocks/Bonds **Jason A. Gluck, DO, FACC**, Abbott: Honoraria **David P. Nicolau, PharmD**, Allergan: Advisor/Consultant|Allergan: Grant/Research Support|Cepheid: Advisor/Consultant|Cepheid: Grant/Research Support|Merck: Advisor/Consultant|Merck: Grant/Research Support|Pfizer: Advisor/Consultant|Pfizer: Grant/Research Support|Shionogi: Advisor/Consultant|Shionogi: Grant/Research Support|Tetraphase: Advisor/Consultant|Tetraphase: Grant/Research Support|Venatorx: Advisor/Consultant|Venatorx: Grant/Research Support|Wockhardt: Advisor/Consultant|Wockhardt: Grant/Research Support **Joseph L. Kuti, PharmD**, bioMeriuex Inc.: Grant/Research Support|Entasis Therapeutics: Grant/Research Support|Merck & Co, Inc: Grant/Research Support|Shionogi Inc: Advisor/Consultant|Shionogi Inc: Grant/Research Support|Shionogi Inc: Honoraria

